# Factors determining the density of AQP4 water channel molecules at the brain–blood interface

**DOI:** 10.1007/s00429-016-1305-y

**Published:** 2016-09-15

**Authors:** Eystein Hellstrøm Hoddevik, Faraz Hameed Khan, Soulmaz Rahmani, Ole Petter Ottersen, Henning Bünsow Boldt, Mahmood Amiry-Moghaddam

**Affiliations:** 1 Division of Anatomy, Department of Molecular Medicine, Institute of Basic Medical Sciences, University of Oslo, Post box 1105, Blindern, 0317 Oslo, Norway; 2 Department of Pathology, Oslo University Hospital, Oslo, Norway

**Keywords:** Astrocyte heterogeneity, Pericyte, Aquaporin-4, α-Syntrophin, Immunogold histochemistry

## Abstract

Perivascular endfeet of astrocytes are enriched with aquaporin-4 (AQP4)—a water channel that is critically involved in water transport at the brain–blood interface and that recently was identified as a key molecule in a system for waste clearance. The factors that determine the size of the perivascular AQP4 pool remain to be identified. Here we show that the size of this pool differs considerably between brain regions, roughly mirroring regional differences in *Aqp4* mRNA copy numbers. We demonstrate that a targeted deletion of α-syntrophin—a member of the dystrophin complex responsible for AQP4 anchoring—removes a substantial and fairly constant proportion (79–94 %) of the perivascular AQP4 pool across the central nervous system (CNS). Quantitative immunogold analyses of AQP4 and α-syntrophin in perivascular membranes indicate that there is a fixed stoichiometry between these two molecules. Both molecules occur at higher densities in endfoot membrane domains facing pericytes than in endfoot membrane domains facing endothelial cells. Our data suggest that irrespective of region, endfoot targeting of α-syntrophin is the single most important factor determining the size of the perivascular AQP4 pool and hence the capacity for water transport at the brain–blood interface.

## Introduction

The water channel aquaporin-4 (AQP4) occurs in abundance at the brain–blood interface and may well be the most plentiful membrane molecule at this site, forming distinctive, orthogonal arrays that make up a substantial areal fraction of the perivascular endfoot membrane (Amiry-Moghaddam and Ottersen 2003; Nielsen et al. 1997; Rash et al. 1998). These arrays were identified in freeze fracture preparations long before AQP4 was cloned and characterised (Dermietzel 1973; Landis and Reese 1974; Rash et al. 1974). Orthogonal arrays are absent from *Aqp4*−/− mice (Verbavatz et al. 1997) and are formed in oocytes transfected with AQP4 cDNA (Yang et al. 1996), bolstering the notion that these arrays are made up of AQP4.

The perivascular pool of AQP4 serves a number of important functions. Mimicking the effects of global AQP4 knockout (Manley et al. 2000; Papadopoulos et al. 2004), a selective depletion of perivascular AQP4 is rate limiting for water flux in the build-up phase and resolution phase of brain oedema (Amiry-Moghaddam et al. 2003a, 2004; Nakayama et al. 2016; Vajda et al. 2002). The perivascular AQP4 pool is critically involved in osmosensing and volume control (Benfenati et al. 2011; Jo et al. 2015; Mola et al. 2016). Recent studies suggest that perivascular AQP4 also plays a pivotal role in the clearance of waste products from brain, possibly by propelling a paravascular drainage route (Iliff et al. 2012). In addition, AQP4 has been implicated in K+ homeostasis and regulation of excitability (Amiry-Moghaddam et al. 2003b; Binder et al. 2004; Haj-Yasein et al. 2012), and loss of perivascular AQP4 precedes chronic seizures in the kainate model of temporal lobe epilepsy (Alvestad et al. 2013). Mislocalisation of AQP4 or changes in the expression level of this water channel have been reported in hippocampi resected from patients with mesial temporal lobe epilepsy (Eid et al. 2005), in mouse models of Alzheimer’s disease (Yang et al. 2011), and in several other pathological and experimental conditions where homeostatic functions are lost or compromised (Nagelhus and Ottersen 2013; Promeneur et al. 2013).

Given the pivotal role of perivascular AQP4 in brain physiology and pathophysiology the question arises as to how the size of this AQP4 pool is determined. There is no unequivocal evidence of AQP4 channel gating (Nagelhus and Ottersen 2013). Thus, current data favour the idea that the water transport capacity at the brain–blood interface is tightly coupled to the number of AQP4 molecules at this site. Here we show that the density of perivascular AQP4 channels varies considerably between regions, roughly matching regional differences in the abundance of *Aqp4* mRNA. All regions showed a substantial loss of perivascular AQP4 following targeted deletion of α-syntrophin. This loss was fairly constant across the central nervous system (CNS), ranging from 79 % in the spinal cord to 94 % in the neocortex. Consistent with these data, quantitative immunogold analyses pointed to a fixed stoichiometry between AQP4 and α-syntrophin in perivascular membranes. Our findings provide new insight in the factors that regulate the size of the perivascular AQP4 pool and hence the capacity for water transport and waste clearance in brain.

## Methods

### Animals

We used adult male and newborn C57BL/6 mice (Jackson Laboratories, Boulder, CO) and two previously described transgenic mouse strains. *Aqp4*−/− animals were generated as described (Thrane et al. 2011) and backcrossed 10 times on a C57BL/6 background; *α*-*syntrophin*−/− (*α*-*Syn*−/−) animals were generated as described (Adams et al. 2000). The mice were allowed ad libitum access to food and drinking water. Animal experiments were performed according to the European Council law on protection of laboratory animals, with the approval of the University of Oslo’s Animal Care and Use Committee. Every effort was made to minimise the number of animals.

### Antibodies

A list of all antibodies used in this study is shown in Table 1.

**Table 1:** Antibodies

Method	Primary antibody	Secondary antibody
Immunogold	AQP4, host: rabbit,Sigma A5971, 1,0 mg/ml diluted 1:400 α-syntrophin (SYN259), host: rabbit, 6 mg/ml diluted 1:200, Gift from Dr. Marvin E. Adams	Goat anti-rabbit 15 nm, Abcam 1:20 Goat anti-rabbit 15 nm, Abcam 1:20
Immunofluorescence	AQP4, host: rabbit,Sigma A5971, 1,0 mg/ml diluted 1:400	Alexa fluor 488, donkey anti-rabbit, Invitrogen, A21206
Immunoperoxidase	AQP4, host: rabbit, Sigma A5971; 1,0 mg/ml diluted 1:400	Biotin Donkey anti-rabbit IgG 31821, Pierce, Thermo Scientific, 1:100 Streptavidin-biotinylated HRP, RPN1051 V, GE Healthcare, 1:100
Western blot	AQP4, anti-rabbit,Sigma A5971; 1,0 mg/ml diluted 1:5000 Actin, anti-rabbit, Sigma, A2066; 0,67 mg/ml diluted 1:200	Mouse monoclonal anti-Rabbit IgG-alkaline phosphatase, A2556, 1:5000

### Transcardial perfusion fixation

Adult animals were anaesthetised with a single intraperitoneal injection of Equithesin (150 μL) and transcardially perfused using 4 % formaldehyde in 0.1 M phosphate buffer (PB) for 15 min with an initial 20 s perfusion with ice cold 2 % dextran in 0.1 M PB. Prompt neck stiffness was used as an indication of satisfactory perfusion. Brains were removed, left in the fixation solution overnight and stored in a 1:10 dilution of the same solution in 0.1 M PB. For newborn (P0) animals, both perfusion velocity and anaesthesia dosage were titrated according to weight and animals were initially sedated using chloroform gas. We employed a modified version of the pH-shift perfusion technique (Eilert-Olsen et al. 2012), initially defined by Berod et al. (1981), for those animals processed further for high resolution electron microscopy to obtain better ultrastructure and minimise epitope loss. This allowed for one half of the brain being used for light and immunofluorescence microscopy while the other was used for embedding in Lowicryl resin.

### Post-embedding immunogold electron microscopy

Brains (*n* = 4 per group, *n* = 2 for each specificity control) were cut into 0.5–1.0 mm slices, regions were dissected, cryoprotected, quick-frozen in liquid propane (−170 °C), and subjected to freeze substitution. Specimens were embedded in methacrylate resin (Lowicryl HM20) and polymerised by UV light below 0 °C. Ultrathin sections (70–100 nm) were cut using an Ultratome (Reichert Ultracut S, Leica) and placed on 300 mesh grids. Immunogold labelling was carried out as previously described (Promeneur et al. 2013). Briefly, sections were rinsed in Tris-buffered saline with Triton X-100 (TBS-T; 5 mM Tris–HCl, 0.3 % NaCl, 0.1 % Triton X-100), incubated in 2 % human serum albumin (HSA), followed by primary antibody (anti-AQP4 or anti-α-syntrophin) overnight, secondary antibody (15 nm gold) for 90 min, and contrasted with 2 % uranyl acetate for 90 s and 0.3 % lead citrate for 90 s. The sections were examined using a Tecnai 12 electron microscope at 80 kV. The examiner was blinded for animal genotype. Appropriate knockout tissue was used as negative control (*Aqp4*−/− and *α*-*Syn*−/− mice) for the two immunogold experiments.

A list of the regions dissected and analysed in this study is shown in Table 2.

**Table 2:** Analysed CNS regions according to experimental assay

Method	Analysed regions and subregions
Immunogold histochemistry	Cortex; parietofrontal cortex, piriform cortex
	Hippocampus; stratum pyramidale, stratum radiatum, dentate gyrus
	Inferior colliculus
	Cerebellum; molecular layer, granular layer, white matter
	Spinal cord (thoracic); white matter, grey matter
Quantitative RT-PCR	Parietofrontal cortex, hippocampus, inferior colliculus, cerebellum, whole brain samples
Western blot	Parietofrontal cortex, inferior colliculus

### Immunogold quantitation

Quantitative analysis was performed as previously described (Amiry-Moghaddam et al. 2004; Lunde et al. 2015). Briefly, images of 20–30 capillaries were acquired from each subregion present on each section. Care was taken to acquire images so that a similar distance of astrocyte membrane abutting on endothelium and pericytes was shown on each picture. We defined inclusion and exclusion criteria for capillaries, astrocyte endfeet and pericytes prior to image collection. In particular, a pericyte was defined as a perivascular cell surrounded by a clearly defined ad- and abluminal basal lamina. Linear densities of gold particles over astrocyte membranes were determined by an extension of analysis [Soft Imaging Systems (SIS), Münster, Germany]. Linear densities were determined semi-automatically and transferred to SPSS Version 22 (SPSS, Chicago, IL) for statistical analysis.

### Immunofluorescence

Mouse brains (*n* = 4 per study group, other half of brains processed for electron microscopy) were cryoprotected in increasing levels of saccharose dissolved in 0.1 M PB (10–20–30 %), and snap frozen in 30 % saccharose on a freeze microtome (Leica). Parasagittal brain sections (30 μm) were cut and collected in wells of sterile cell culture plates containing 0.4 % PFA diluted in 0.1 PB and stored in cold room (4 °C). For immunofluorescence, sections were washed with 0.01 M phosphate buffered saline (PBS) and permeabilised for 10 min with 0.1 % Triton X-100 in PBS. After blocking with 2 % bovine serum albumin (BSA) in PBS, sections were incubated overnight with primary antibodies diluted in blocking solution. Following three washes with PBS, sections were incubated with secondary antibodies diluted in blocking solution for 90 min. After a further wash with PBS, sections were incubated for 5 min with a Hoechst preparation, thereafter washed with PBS and mounted using Prolong Gold antifade reagent (Thermo Fisher). Confocal images were acquired using a Leica fluorescence microscope.

### Immunoperoxidase

Mouse brains (*n* = 3 per group) were cryoprotected and sectioned as described above. Sections were washed with PBS, exposed to 2 % H_2_O_2_ for 10 min to deplete endogenous peroxidase activity, and washed three times with PBS. Blocking was done using 2 % BSA in PBS-T (PBS, 0.1 % Triton X-100). Incubation with primary antibody diluted in blocking buffer was done overnight in cold room (4 °C), followed by three washes with PBS-T. A 1 h incubation with secondary antibody in blocking buffer ensued, followed by three washes with PBS-T, incubation for 1 h with biotinylated-streptavidin horseradish peroxidase complex (GE healthcare UK limited, Rpn 1051 V) diluted 1:100 in PBS-T, three washes with PBS-T, then three washes with PBS. Sections were then incubated for 5 min with 0.5 mg/mL diaminobenzidine (3,3-diaminobenzidine tetrahydrochloride 10 mg/tab, Sigma D5905, DAB) dissolved in 0.1 M PB and exposed to DAB solution containing 0.01 % H_2_O_2_ (90 s). The DAB/HRP reaction was stopped by three washes with 0.1 M PB. Sections were mounted using glycerine gelatine, scanned using a Mirax scanner (Zeiss) and examined using a light microscope.

### Lysates and tissue processing for Western blotting and quantitative RT-PCR

WT and *α*-*Syn*−/− mice were anaesthetised with isofluorane and decapitated. Brains were dissected out and split in two with a midline, sagittal cut. Regional dissections (*n* = 4) and whole brain samples (*n* = 4) were processed for quantitative RT-PCR analysis by overnight incubation in RNAlater (Ambion) and storage at −80 °C until further processing. Regional dissections (*n* = 4) from parietal cortex and colliculus inferior were obtained and processed for western blotting. Samples were put into 2 mL Lysing matrix D (MP Biomedicals) Eppendorf tubes containing RIPA (radio-immunoprecipitation assay) homogenisation buffer with protease inhibitor cocktail (Roche) added immediately before use, samples were homogenised in a FastPrep FP120 Cell Disrupter (MP Biomedicals), shaken at intervals and intermittently cooled on ice. Supernatant of a 9300 × g spin for 30 min at 4 °C was used for immunoblotting; pellets were frozen at −80 °C. Protein concentrations were determined with a DC protein assay (Bio-Rad) according to the manufacturer’s instructions. Equal numbers of right and left hemispheres were included in each analysis.

### Western blot

Gels were cast using the Mini-Protean III cell from Bio-Rad as previously described (Sorbo et al. 2007). Both stacking and resolving gels were cast with added 3 M urea. 12 % SDS-PAGE gels were run at 150 V for 60 min, blotted with the Criterion cell (Bio-Rad), equilibrated with Towbin buffer (25 mM Tris, 192 mM glycine) supplemented with 20 % methanol and blotted onto 0.2 μm pore size PVDF membranes (Bio-Rad) at 100 V for 40 min. Blots were rinsed briefly in TBS-T (20 mM Tris; 137 mM NaCl; 0.05 % Tween-20 (Sigma), 0.05 % NaN_3_; pH = 7.6) prior to incubation for 30 min with 5 % non-fat dried milk powder (Applichem) dissolved in TBS-T (blocking buffer). Blots were incubated with rabbit anti-AQP4 (Sigma) diluted in blocking buffer overnight at 4 °C, then washed in TBS-T, incubated in a 1:5000 dilution in TBS-T of alkaline phosphatase conjugated to mouse monoclonal anti-Rabbit IgG (A2556 Sigma) for 1 h at room temperature and subsequently washed in TBS-T for 3 h. Finally, blots were incubated for 15 min with the ECF substrate (Amersham, ECF Kit), rinsed briefly in TBS-T, dried and scanned with a Kodak desktop scanner (Kodak Image Station 4000 MM PRO). Certain blots were re-probed with an anti-actin antibody (Sigma). Briefly, blots were immersed in methanol, incubated with blocking buffer for 30 min, then incubated with anti-actin diluted in blocking buffer at 4 °C overnight, rinsed and scanned as described above for the initial primary antibody. All samples were heat treated for 10 min at 37 °C, spun at 13,000 rpm for 10 min, resuspended and loaded onto the gel. All samples were assayed for total protein content using the DC-kit (Bio-Rad) with BSA diluted in H_2_O as standard. 5 μg of protein was loaded in each well, except for control tissue (*Aqp4*−/−) where 10 μg was added. Precision plus protein standards Dual colour (Bio-rad) was used as Mw ladder.

### Quantitative RT-PCR

Quantitative RT-PCR was carried out as described previously (Lunde et al. 2015). In brief, total RNA was extracted using RNeasy Lipid Tissue kit (Qiagen). 2 μg of RNA from whole brain and 100–1000 ng of RNA from regions were reverse-transcribed using oligo d(T)18 primer and RevertAid H Minus First Strand cDNA transcription reagents (Fermentas). The real-time PCR was set up using 2 μL template, corresponding to 5 ng cDNA, in a reaction volume of 20 μL containing AB Power SYBR Green PCR Master Mix (Applied Biosystems) and primers (200 nM) for detection of mouse *Aqp4*: TTTGGACCCGCAGTTATCAT (forward) and GTTGTCCTCCACCTCCATGT (reverse). Expression was assessed by absolute quantification using the StepOnePlus real-time PCR system (Applied Biosystems). Tbp (ACGGACAACTGCGTTGATTT (forward); CAAGGCCTTCCAGCCTTATAG (reverse)) was used as endogenous control for normalisation of gene expression. SPSS was used for statistical analysis. *T* test was used for comparing *Aqp4* expression in WT and *α*-*Syn*−/− mice. For comparing expression among regions, one-way ANOVA Bonferroni post hoc was used.

## Results

### AQP4 expression displays regional heterogeneity in adult and newborn murine brain

**Fig. 1:**
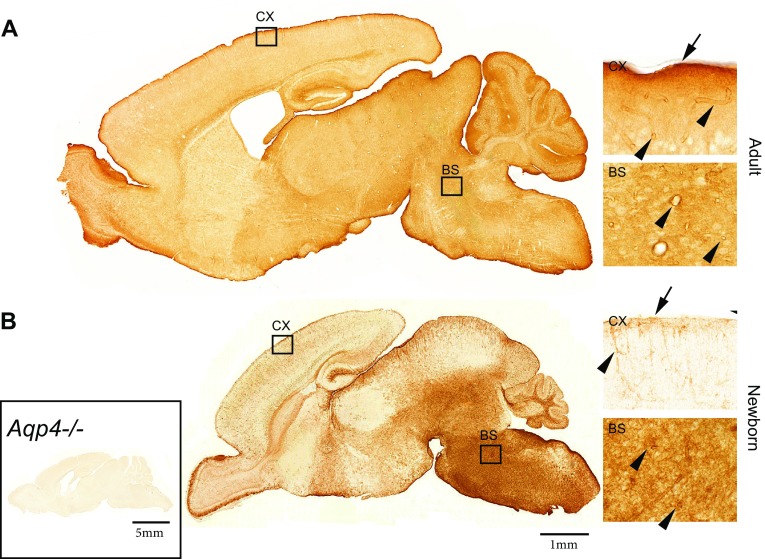
AQP4 expression displays regional heterogeneity in adult and newborn mouse brain. Sagittal sections from an adult (**a**) and a newborn (**b**) mouse brain demonstrate distinct regional differences in AQP4 immunoperoxidase labelling intensity. In both sections, neocortex and striatum demonstrate the least pronounced staining. Heterogeneity in labelling intensity is seen in a caudo-rostral and ventro-dorsal developmental pattern which is already established at birth (**b**). *Insets* (*right panel*) show higher magnification of cortex (*CX*) and brainstem (*BS*) for both ages. Cortex in adult mice exhibit distinct labelling of glia limitans (*arrow*) and of vasculature (*arrowheads*). The overall labelling intensity is less pronounced in the newborn cortex, where the labelling is located mainly at the glia limitans (*arrow*) and vessels (*arrowheads*) close to the brain surface. The strong labelling observed in newborn brainstem and cerebellar white matter is located primarily in brain parenchyma, and to a much lesser extent around blood vessels (*arrowheads*). Specificity control (*left inset*, *Aqp4*−/−) is shown on a sagittal section from an adult *Aqp4*−/− mouse where no residual labelling is seen

Light microscopic immunohistochemistry shows distinct caudo-rostral and ventro-dorsal gradients in AQP4 immunolabelling. Thus, labelling is notably stronger in cerebellum, olfactory bulb, spinal cord and brainstem than in cerebral cortex and striatum (Fig. 1). In the adult brain, AQP4 labelling is concentrated around blood vessels and subjacent to pia. A regional heterogeneity in AQP4 distribution is evident in the adult brain (Fig. 1a) as well as postnatally (Fig. 1b; also see Lunde et al. 2015). However, at this early stage the AQP4 immunolabelling is not concentrated around vessels or subpially, but rather evenly distributed in the neuropil. Absence of labelling in *Aqp4*−/− mice attests to the selectivity of the immunolabelling (Fig. 1b inset).

### Evidence of non-uniform expression of AQP4 in pericapillary endfeet

**Fig. 2:**
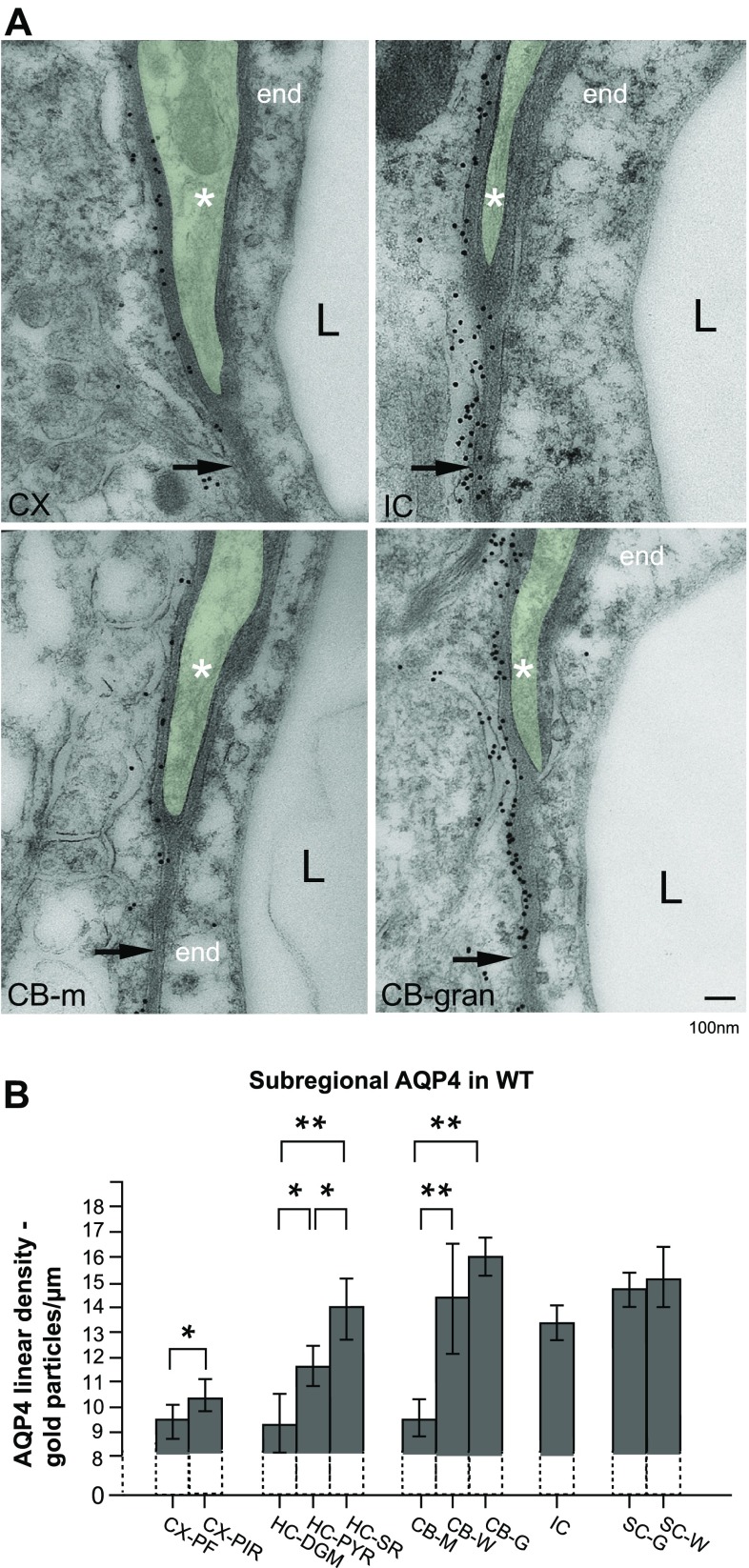
Regional and subregional perivascular heterogeneity of AQP4 immunogold labelling. Shown in **a** are portions of perivascular structures and cells from four CNS subregions. Each electron microscopic picture shows endothelium (end), the capillary lumen (*L*) and part of a pericyte (*coloured green* and annotated with *asterisk*) surrounded by a basal lamina on both sides. Immunogold particles can be seen along the length of the perivascular astrocyte endfoot membrane, but fewer gold particles per membrane length are seen in the membrane abutting endothelium (*arrow*) compared to the membrane domain abutting pericytes. Perivascular labelling is notably less intense in the endfoot from cortex (CX, *upper left*) compared to that of the inferior colliculus (IC, *upper right*). This heterogenic pattern is also present in layers within the same region, shown here for cerebellum (CB-m, CB-gran, *lower panel*), whereby immunogold linear density of granular cell layer is remarkably more pronounced than in cerebellar molecular layer. **b** Adjacent subregions within the hippocampal (HC-PYR; stratum pyramidale, HC-SR; stratum radiatum, HC-DGM; molecular layer of the dentate gyrus) and cerebellar formation (CB-G; cerebellar granular layer, CB-W; cerebellar white matter, CB-M; cerebellar molecular layer) demonstrate statistically significant differences in mean linear AQP4 density when compared within each region. In cerebellum, AQP4 density of the molecular layer (mean 9.45) is considerably lower than that of both granular layer (mean 15.84) and white matter (14.23). Within the hippocampal formation, perivascular immunogold density was significantly less pronounced in dentate gyrus (mean 9.24) than in stratum pyramidale (11.59) and stratum radiatum (13.86). Parietofrontal cortex (CX-PF) also exhibits weaker labelling than piriform cortex (CX-PIR). No statistically significant difference was found between *white* (SC-W, mean 15.11) and *grey* (SC-G, mean 14.68) matter of spinal cord. *Bar* 100 nm. * and ** show *p* < 0.05 and *p* < 0.001, respectively. *Error bars* represent 95 % confidence interval

Quantitative, high-resolution AQP4 immunogold histochemistry on ultrathin sections confirms and expands observations from light microscopy. AQP4 is found to be constitutively expressed in pericapillary endfeet and enriched in the endfoot membranes where levels display significant regional variation (Fig. 2a). Cortex (CX) and molecular layer of cerebellum (CB-m) display sparse labelling in contrast to the intense labelling of regions such as inferior colliculus (IC) and cerebellar granular layer (CB-gran). Linear density of gold particles along perivascular endfoot membranes was determined for capillaries of cortex, hippocampus, cerebellum, inferior colliculus and spinal cord and provides evidence of significant region-specific variations. Heterogeneity is also present within subregions of the same anatomical region, shown here for hippocampus and cerebellum (Fig. 2b). No statistically significant difference could be found between grey and white matter regions of the spinal cord. In cerebellum, AQP4 density in the molecular layer is considerably lower than that in the granular layer and white matter. Within the hippocampal region, perivascular immunogold density is significantly lower in dentate gyrus than in stratum pyramidale and stratum radiatum. When the immunogold data is grouped according to region, the spinal cord and neocortex show the highest and lowest mean linear densities, respectively (14.80 vs. 9.82 particles per um; Fig. 4a, left, b, top).

### Evidence of an α-syntrophin independent pericapillary pool of AQP4

**Fig. 3:**
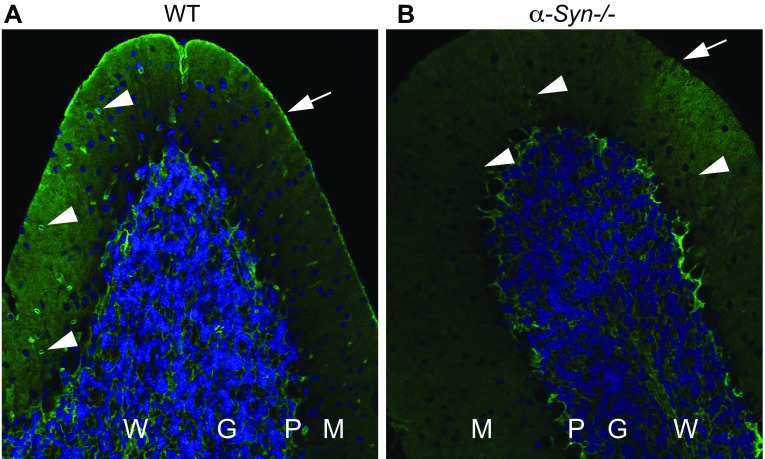
Impact of α-*syntrophin* gene deletion on AQP4 distribution. Immunofluorescence micrographs of cerebellar folia (×20 magnification) from a WT (**a**) and α-*Syn*−/− mouse (**b**) are shown, depicting the molecular (*M*), Purkinje (*P*), granular (*G*) and white matter (*W*) layers of the cerebellum. *Both images* show AQP4 immunostaining (*green*) and nuclear DAPI staining (*blue*). Genetic deletion of *α*-*syntrophin* leads to loss of perivascular (*arrow head*) and subpial (*arrow*) AQP4 labelling (*arrow head*). Staining of astrocyte processes surrounding Purkinje cells appears more intense in sections from *α*-*Syn*−/− mice (**b**) compared to WT controls (**a**)

**Fig. 4:**
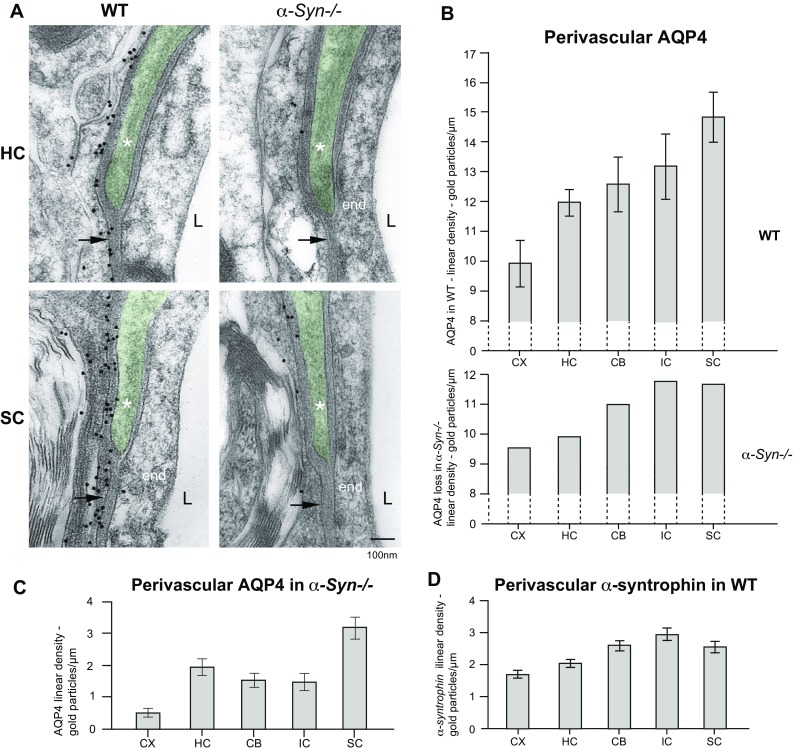
Regional perivascular heterogeneity is retained in *α*-*Syn*−/− mice. **a** Perivascular AQP4 immunogold labelling in WT and *α*-*Syn*−/− mice is illustrated by representative images from hippocampus (*HC*) and spinal cord (*SC*). *Each picture* shows endothelium (end), the capillary lumen (*L*) and part of a pericyte (*coloured green* and annotated with *asterisk*). For both regions, AQP4 labelling intensity is dramatically reduced in *α*-*Syn*−/− mice compared to controls (*WT*) but some residual gold particles are observed. **b** *Diagram* (*top*) showing quantitation of AQP4 mean linear density in perivascular astrocyte domains for five brain regions. Significant differences in perivascular AQP4 labelling between the examined regions can be seen: cortex displays the lowest density (mean 9.82), followed by hippocampus (11.82), cerebellum (12.53), inferior colliculus (13.22) and spinal cord (14.80). Cortex has a statistically significant lower density when compared to the other regions (*p* < 0.001) and the density in spinal cord is significantly higher compared to the other four regions (*p* < 0.001). Following *α*-*syntrophin* gene deletion, a substantial portion of membrane bound AQP4 in endfeet is lost. The calculated mean loss for each region is shown in the *bottom diagram* and appears proportional to the AQP4 density in the WT brain. The relative loss of AQP4 is also less pronounced in spinal cord compared to other regions. **c** *Diagram* showing mean AQP4 linear density from the five quantified regions in *α*-*Syn*−/− mice. All regions in *α*-*Syn*−/− mice lose most of the perivascular AQP4 labelling, but the regional heterogeneity pattern is retained: cortex remains the region of lowest AQP4 linear density (mean 0.58) and spinal cord still displays the highest perivascular labelling (3.16). No statistical difference is found between CB (1.59), HC (2.00) and IC (1.51). **d** Diagram showing quantitative analysis of perivascular α-syntrophin immunogold labelling in five regions. Regional heterogeneity of perivascular α-syntrophin labelling follows that of AQP4. Cortex displays the lowest linear density of α-syntrophin (mean 1.69) followed by HC (2.01), SC (2.52), CB (2.59) and IC (2.91). *Error bars* represent 95 % confidence intervals

Due to prior evidence implicating α-syntrophin as an anchoring molecule of endfoot AQP4 (Neely et al. 2001), we assessed AQP4 localisation in *α*-*Syn*−/− mice. Genetic deletion of α-syntrophin leads to a pronounced reduction in perivascular AQP4 labelling in all regions and subregions analysed, as exemplified by images from cerebellum (Fig. 3). The representative image from an *α*-*Syn*−/− mouse (Fig. 3b) suggests a redistribution of AQP4 whereby perivascular and subpial AQP4 immunostaining in *α*-*Syn*−/− animals is not detectable, but staining of astrocyte processes surrounding Purkinje cells appears more intense than in wild-type mice (Fig. 3a). Immunogold detection of AQP4 is more sensitive than immunofluorescence, and—albeit for the notable reduction (79–94 %)—demonstrates residual pericapillary AQP4 immunogold labelling in *α*-*Syn*−/− animals (Fig. 4a). Immunogold labelling was abolished in control sections from *Aqp4*−/− mice (specificity control, not shown).

The present analysis is restricted to the perivascular labelling. It should be noted, however, that the granular layer of the cerebellum contains a sizeable non-endfoot AQP4 pool that is largely independent of α-syntrophin and dystrophin (Fig. 3, see Amiry-Moghaddam et al. 2004; Nicchia et al. 2008b).

### The size of residual AQP4 around capillaries is subject to regional variations

Residual AQP4 labelling around capillaries was quantified by immunogold histochemistry (Fig. 4a–c). Strikingly, in *α*-*Syn*−/− mice, perivascular AQP4 immunogold linear density in spinal cord is more than fourfold higher than in cortex (Fig. 4c). Following α-syntrophin gene deletion, cortex remains the region of lowest AQP4 linear density (mean 0.58 particles/um) while spinal cord still displays the highest perivascular labelling (3.16 particles/um). There is thus compelling evidence for retained, heterogeneous expression of AQP4 in *α*-*Syn*−/− mice.

### Genetic deletion of α-syntrophin results in elimination of a similar proportion of AQP4 in all endfoot membranes, irrespective of the size of the AQP4 pool at this site

The relative AQP4 loss following *α*-*syntrophin* gene deletion is most pronounced in CX (94 %) followed by IC (89 %), CB (87 %), HC (83 %) and SC (79 %). The nominal loss in AQP4 linear density following α-syntrophin deletion, calculated as difference between means of linear density of AQP4 in knockout vs. wildtype (Fig. 4b), is greatest for IC and SC (11.71 and 11.64 particles/um, respectively), followed by CB (10.96), HC (9.83) and CX (9.25).

### Linear density of perivascular α-syntrophin correlates positively with that of AQP4

Perivascular α-syntrophin immunogold labelling (Fig. 4d) in WT mice shows how α-syntrophin levels correlate positively with that of AQP4 density (Fig. 4b) in endfoot membranes. Capillaries of cortical regions display the lowest density of α-syntrophin followed by hippocampus, cerebellum, spinal cord and inferior colliculus. As illustrated (Fig. 4b, d), the ratio of AQP4 to α-syntrophin varies little between regions. The calculated nominal ratio between AQP4 and α-syntrophin (in terms of immunogold particles) is 5.82 for cortex, 5.89 for hippocampus, 4.84 for cerebellum, 4.54 for inferior colliculus and 5.87 for spinal cord. Labelling was abolished in control sections from *α*-*Syn*−/− mice (specificity control, not shown).

### Quantitative RT-PCR demonstrates regional differences in gene expression

**Fig. 5:**
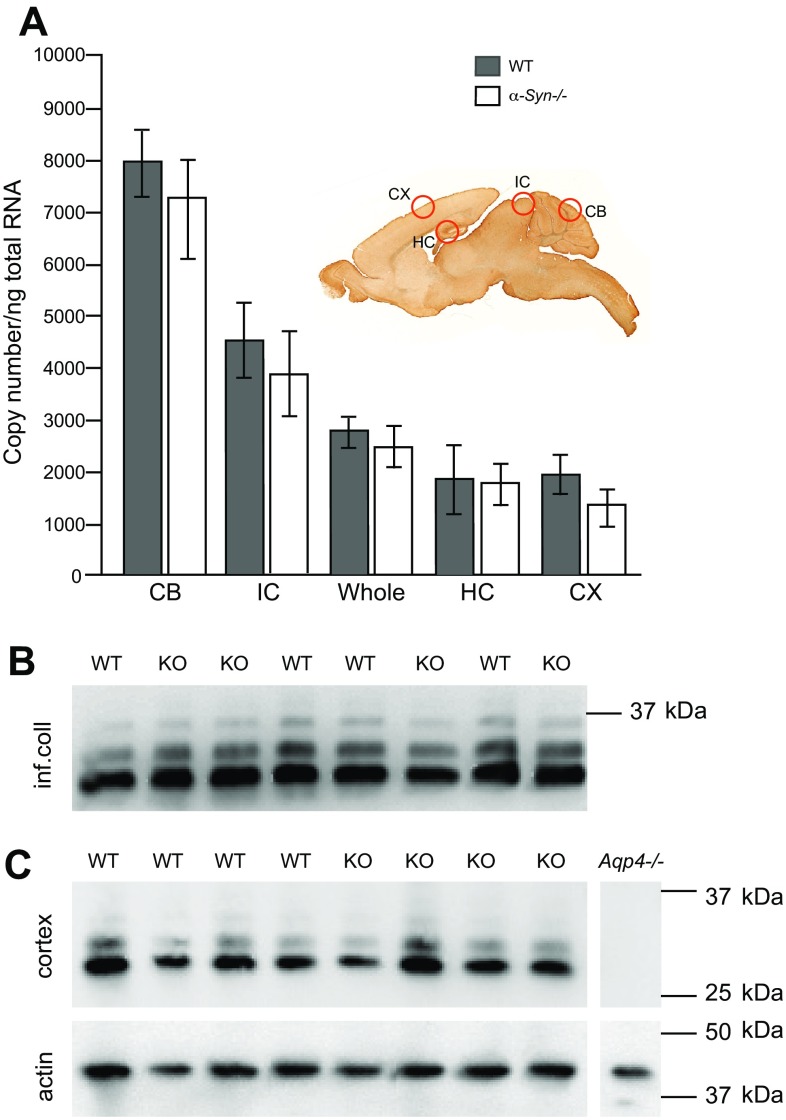
Differential *Aqp4* mRNA expression and AQP4 protein in brain regions from WT and *α*-*Syn*−/− mice. **a** Sagittal section of a mouse brain indicating the regions analysed. qRT-PCR analysis shown in **a** demonstrates that for WT animals, the mean copy number of *Aqp4* mRNA per nanogram of total RNA is the highest in CB (7928) followed by IC (4521), HC (1873) and CX (1920) while mean copy number for whole brain was 2787. For *α*-*Syn*−*/*− animals, the mean copy number of *Aqp4* mRNA is the highest in CB (7115), followed by IC (3876), HC (1783) and CX (1368), mean copy number for whole brain was 2495. No statistically significant difference between the two genotypes is seen for any of the regional dissections, or for the whole brain samples. Cerebellum (*CB*) and inferior colliculus (*IC*) show higher expression of *Aqp4* mRNA than cortex (*CX*) and hippocampus (*HC*). Shown in **b**, **c** (*top*) are western blots showing anti-AQP4 labelling for two brain regions from WT and *α*-*Syn*−*/*− (*KO*) mice. No difference in AQP4 labelling between genotypes is seen for either cortex or inferior colliculus (**b**, **c** *top*). Samples from wild-type and *α*-*Syn*−/− mice (*n* = 4 for each genotype) are included. Two major bands, corresponding to M1 and M23 isoforms of APQ4, are detected in samples from both regions. Samples from inferior colliculus (**b**) consistently display more intense AQP4 immunostaining than those from cortex (**c**, *top*). No labelling is seen in the samples from *Aqp4*−/− mice incubated with anti-AQP4 (specificity control). *Error bars* represent 95 % confidence intervals

Shown in Fig. 5a is quantitative evidence of regional differences in *Aqp4* mRNA for both WT and *α*-*Syn*−/− genotypes. For WT animals, the mean copy number of *Aqp4* mRNA is highest in cerebellum, followed by inferior colliculus, whole brain, then hippocampus and cortex. Similarly, for *α*-*Syn*−/− animals, the mean copy number of *Aqp4* mRNA is highest in cerebellum followed by inferior colliculus, whole brain, hippocampus and cortex. For WT animals, the number of mRNA copies detected in cerebellum is fourfold that detected in cortex (*p* < 0.001), and the amount of mRNA copies detected in inferior colliculus is twice as high as in cortex (*p* < 0.001). We find no statistical difference between hippocampus and cortex in regard to total copy numbers of *Aqp4* mRNA. This is true for WT as well as *α*-*Syn*−/− mice. Notably, there is no difference between *Aqp4* expression in whole brain samples (*p* = 0.188) when comparing WT and *α*-*Syn*−/−. The same holds true for all regions (from CB, IC, HC and CX).

### Western blot analysis of AQP4 shows no difference between genotypes and demonstrates qualitative differences in AQP4 levels of CX and IC

Immunoblots from inferior colliculus (IC) and cortex demonstrate two major bands at the expected molecular weight, corresponding to M1 and M23 isoforms of AQP4, respectively (Fig. 5b, c). Both bands appear stronger in the immunoblot containing homogenates from inferior colliculus (Fig. 5b) compared to those from cortex (Fig. 5c) and provide qualitative data in accordance with the quantitative real-time PCR analysis. Anti-actin immunoblot on cortical samples is included to demonstrate presence of protein in *Aqp4*−/− mice. Importantly, no difference in AQP4 labelling patterns or intensity can be seen between WT and *α*-*Syn*−/− mice, as assessed for cortex and inferior colliculus (Fig. 5b, c). This is in line with earlier studies, indicating that deletion of α-syntrophin causes a mislocalisation rather than a loss of AQP4 from brain tissue (Neely et al. 2001).

### Pericapillary astrocyte endfeet abutting pericytes are enriched in both AQP4 and α-syntrophin

**Fig. 6:**
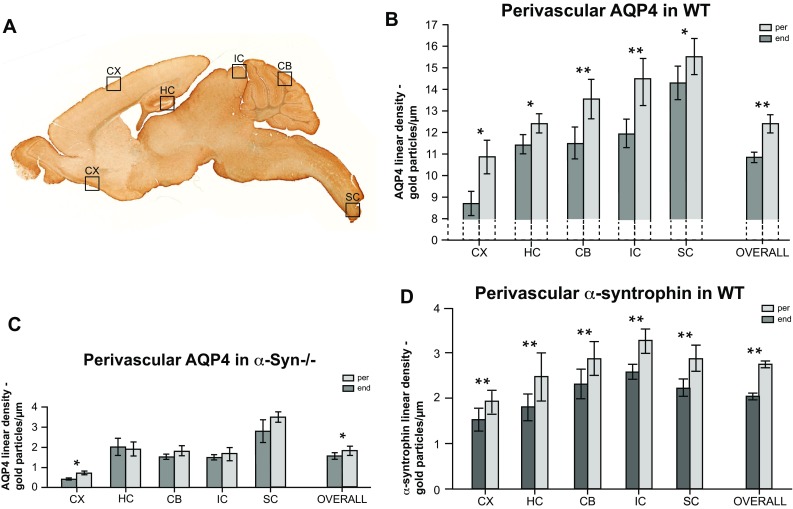
Pericapillary astrocyte endfeet abutting pericytes are enriched in both AQP4 and α-syntrophin. **a** Sagittal section of mouse brain indicating the dissected regions subjected to post-embedding immunogold histochemistry against AQP4 and α-syntrophin. Piriform and parietal cortex were grouped and reported as cortex. **b** Quantitative immunogold analysis demonstrates statistically significant differences in the mean linear density of AQP4 between perivascular astrocyte membrane domains abutting pericytes (per) and endothelium (end) in all the regions examined. To the *far right* is a graphic representation of all regions grouped together, annotated ‘overall’. AQP4 linear density is 15 % higher in endfoot membrane domains abutting pericytes than for endfoot membrane domains abutting endothelium, **c** The overall difference between the AQP4 density in the endfeet abutting pericytes compared to adjacent membranes abutting endothelium is retained in *α*-*Syn*−/− mice. AQP4 linear density is 16 % higher in *per* compared to *end* membrane domains. Within subregions, the difference in AQP4 linear density of astrocyte membranes abutting endothelium compared to those abutting pericytes only reaches statistical significance in cortex. **d** Quantitative analysis of sections from WT animals shows that the linear density of α-syntrophin immunogold particles in astrocyte endfeet membrane domains abutting pericytes is consistently higher than in the endfeet abutting endothelium for all regions examined. *Error bars* represent 95 % confidence intervals. * and ** show *p* < 0.05 and *p* < 0.001, respectively

As measured by quantitative immunogold labelling in WT mice, the overall AQP4 linear density is 15 % higher for endfoot membrane domains abutting pericytes than for endfoot membrane domains abutting endothelium (Fig. 6b, *p* < 0.001). This enrichment is statistically significant and present in all examined regions (*p* < 0.05 for CX, HC, SC and *p* < 0.001 for CB and IC), ranging from a factor of 1.26 higher in cortex to 1.08 in spinal cord. Nominally, the difference in linear density ranges from 2.60 (IC) to 1.01 (HC) gold particles per μm. We next investigated to what extent the regional, non-uniform expression of AQP4 in pericapillary endfeet was dependent on α-syntrophin. The difference between adjacent membrane domains remains following α-syntrophin gene deletion. Overall AQP4 linear density is 16 % higher in endfeet abutting pericytes compared to adjacent membranes abutting endothelium (Fig. 6c, *p* < 0.05) in *α*-*Syn*−/− animals. The nominal difference of linear density between these two membrane domains is decreased in *α*-*Syn*−*/*− mice, however, and a statistically significant difference (*p* < 0.05) was only observed in CX (Fig. 6c). As is the case for AQP4, α-syntrophin is also enriched in endfoot domains abutting pericytes compared to those abutting endothelium, a difference which reaches statistical significance for all examined regions (Fig. 6d, *p* < 0.001). The relative difference in α-syntrophin linear density between pericyte and endothelium abutting endfoot membrane domains is constant across examined regions, by a factor ranging from 1.27 (CB) to 1.38 (HC).

## Discussion

Given the emerging evidence pointing to the involvement of pericapillary AQP4 in a range of brain functions and pathologies there is a need to identify the factors that determine the size of this pool of AQP4. We decided to use the following, two step approach: first, we assessed the range by which the perivascular density of AQP4 varies across regions and subregions. Second, we aimed to identify the molecular basis of this heterogeneity.

The present study is restricted to the pericapillary AQP4 pool since this is the one that is most directly involved in regulating water flux across the brain–blood interface. Other AQP4 pools in brain (perisynaptic and subpial) have been investigated in previous studies (reviews: Amiry-Moghaddam and Ottersen 2003; Manley et al. 2004; Nagelhus and Ottersen 2013). It should also be emphasised that no attempt was made to discriminate between the two main isoforms of AQP4—M1 and M23. Thus, even if they differentially engage in supramolecular assemblies (square arrays, Furman et al. 2003; Hiroaki et al. 2006; Nicchia et al. 2008a), these two isoforms flux water at the same rate (Jung et al. 1994), The AQP4 antibody presently used is directed to the C-terminus and recognises isoform M1 as well as M23.

Little is known about the extent to which the perivascular AQP4 pool differs in size between regions and subregions. In the only study that has addressed this issue thus far (Amiry-Moghaddam et al., 2004) we showed that the perivascular pool of AQP4 increased in size on moving from the molecular layer to the granular cell layer of the cerebellum. This study left us with several important questions that now emerge as highly important and relevant, given the recent breakthroughs in our understanding of water transport in brain. In particular, the notion that perivascular AQP4 might be a key element in the brain’s waste clearance system has drawn much attention and calls for new analyses (Igarashi et al. 2014; Iliff et al. 2012; Nagelhus and Ottersen 2013; Papadopoulos and Verkman 2013).

First, we ask whether the differences that were uncovered within the cerebellum (Amiry-Moghaddam et al. 2004) are a reflection of large scale differences between regions and subregions in the brain. Second, we ask whether such differences are rooted in differential transcription rates of the *Aqp4* gene and/or differential targeting of α-syntrophin to perivascular endfeet. It has long been known that α-syntrophin contributes to the anchoring of the perivascular AQP4 pool (Neely et al. 2001). However, it remains to be explored whether any heterogeneity in perivascular AQP4 expression can be attributed to differential expression of α-syntrophin.

### Regional differences in the abundance of endfoot AQP4 imply regional differences in water transport capacity

Based on our quantitative immunogold data, we conclude that perivascular endfoot membranes differ considerably with respect to the amount of AQP4 they contain. Differences occur between regions and subregions, as well as on the level of individual capillaries (with endfoot membranes facing pericytes containing more AQP4 than endfoot membranes facing endothelia). This suggests that there is a corresponding non-uniformity in the capacity of water transport. AQP4 is the only aquaporin that is known to be concentrated in endfoot membranes. However, there is not necessarily a strict correlation between pericapillary AQP4 expression and water transport capacity. Thus, we cannot rule out the possibility that AQP4 is differentially regulated, depending on region, subregion or membrane microdomain, Several factors have been identified that affect water flux through AQP4 (Yukutake et al. 2008; Yukutake and Yasui 2010; Zelenina 2010) although direct evidence for bona fide gating is still missing (Nagelhus and Ottersen 2013). Available evidence suggests that the capacity for water transport at the brain–blood interface determines the propensity for oedema formation as well as the capability for oedema resolution (Amiry-Moghaddam et al. 2003a; Papadopoulos et al. 2004; Zeynalov et al. 2008).

### Evidence for a stoichiometric coupling of AQP4 and α-syntrophin

By use of quantitative immunogold analyses in combination with genetic manipulation we showed that a major fraction of the AQP4 pool in endfoot membranes is dependent on the presence of α-syntrophin. While it has long been known that α-syntrophin serves as a molecular anchor of endfoot AQP4 (Neely et al. 2001), this study is the first to show that the densities of α-syntrophin and AQP4 appear to be stoichiometrically coupled. Thus, the nominal ratio between the two molecules in terms of their immunogold labelling intensities varies little among regions (4.54 for inferior colliculus, 4.84 for cerebellum, 5.82 for cortex, 5.87 for spinal cord and 5.89 for hippocampus). Obviously, this ratio does not necessarily reflect the actual molecular ratios, as AQP4 and α-syntrophin may differ in regard to their labelling efficiency (defined as number of gold particle per target molecule). A more direct assessment of the actual molecular ratio (through biochemical approaches) must await the development of techniques for selective isolation of endfoot membranes. However, the uniformity in terms of AQP4/α-syntrophin gold particle ratios is consistent with the idea that the complement of AQP4 in the endfoot membrane is governed by the number of α-syntrophin molecules that are targeted to this membrane.

### Targeted disruption of the α-syntrophin gene deletes a similar fraction of the astrocyte endfoot pool of AQP4, irrespective of brain region

Our finding of a fairly uniform AQP4/α-syntrophin ratio does not necessarily imply that α-syntrophin is the only anchoring molecule for AQP4. Indeed, confirming previous observations (Amiry-Moghaddam et al. 2004), we show that there is a residual pool of the astrocyte endfoot AQP4 following targeted deletion of α-syntrophin. Here we extend these observations by demonstrating that the size of the residual pool varies in proportion to the amount of AQP4 in WT endfeet. In other words, the percentage of perivascular AQP4 removed by deletion of α-syntrophin does not differ significantly between regions. The most probable explanation is that the residual AQP4 pool is anchored by a molecule whose expression is proportional to that of α-syntrophin. This unknown anchoring protein may well be another syntrophin, such as β-syntrophin (Puwarawuttipanit et al. 2006). This hypothesis will be explored once β-syntrophin knockout animals are made available.

α-Syntrophin and β-syntrophin are parts of a multimember molecular assembly—the dystrophin associated protein complex (DAPC; Frigeri et al. 2001; Neely et al. 2001). Mdx mice that lack the brain dystrophin DP71 also lack a sizeable endfoot pool of AQP4 (Frigeri et al. 2001; Vajda et al. 2004). The DAP complex is held in place through its attachment to laminin (Culligan et al. 2001; Neely et al. 2001). This explains why there is such a sudden drop in AQP4 as soon as the endfoot plasma membrane is reflected away from the capillary.

In line with the important role of DAPC in AQP4 anchoring, deficient *O*-glycosylation of α-dystroglycan (a member of DAPC) causes a reduction in perivascular AQP4 (Michele et al. 2002; Rurak et al. 2007). Further, agrin (which interacts with α-dystroglycan) is required for normal expression of AQP4 in brain endfeet. It is important to note, however, that the brain also contains DAPC-independent pools of AQP4. One such pool is found in cerebellar astrocytes Nicchia et al. 2008b). This complexity notwithstanding, the present data show that α-syntrophin is a major player in controlling the expression of AQP4 at the brain–blood interface.

### The level of AQP4 mRNA covaries with the level of perivascular AQP4

Our quantitative RT-PCR analysis revealed pronounced regional differences in the amount of *Aqp4* mRNA. The *Aqp4* transcript levels were high in those regions that displayed high levels of AQP4 in perivascular endfeet and were not sensitive to α-syntrophin deletion.

### Heterogeneous distribution of AQP4 within individual endfoot membranes

Superimposed on the regional differences in the size of the endfoot AQP4 pool there is a heterogeneity in AQP4 distribution within individual endfoot membrane domains. Specifically, the densities of AQP4 and α-syntrophin immunogold particles are higher where endfoot membranes abut pericytes than where they abut endothelium. This is true not only for cortical astrocytes (Gundersen et al. 2013) but for all regions included in the present analysis. The local increase in the amount of AQP4 and α-syntrophin could be a direct effect of pericytes—through secretion of soluble factors or extracellular matrix molecules. As pericytes are contractile, they could also affect contiguous membranes mechanically. This is of relevance, as a recent cell-culture study from our laboratory showed increased AQP4 levels in astrocyte processes subjected to mechanical stress (Camassa et al. 2015).

### The mechanistic basis for the non-uniform expression of AQP4 in astrocyte endfeet

Taken together, our data suggest that α-syntrophin is a key factor in regulating the AQP4 pool of perivascular endfoot membranes. Drawing on quantitative, high resolution immunogold analyses, we provide evidence that the number of AQP4 molecules is stoichiometrically coupled to that of α-syntrophin. A definition of the exact molecular ratio between these molecules must await the development of appropriate techniques for isolating endfoot membranes. That AQP4 and α-syntrophin covary is not a coincidental correlation but reflects a causality: removal of α-syntrophin via genetic deletion removes a substantial fraction of endfoot AQP4 that varies little across regions. What determines the size of the AQP4 pool and hence the endfoot’s water fluxing capacity, therefore, is the number of α-syntrophin molecules that are targeted to the endfoot membrane. The alternative explanation—that α-syntrophin regulates *Aqp4* expression through regulation at the transcriptional level—is ruled out by our finding that the number of *Aqp4* mRNA copies remains constant in *α*-*syntrophin* knockout animals.

Our experiments show that the level of AQP4 in endfeet is correlated to the level of *Aqp4* mRNA in the corresponding region. This observation does not necessarily imply that the level of endfoot AQP4 is controlled at the transcriptional level. Thus, the transcriptional regulation of *Aqp4* is complex (Pisani et al. 2011; Rossi et al. 2010), requiring further studies.

## Conclusion

Three seminal findings have provided new insight in the mechanisms that control the size of the perivascular AQP4 pool, and thus the capacity for water transport and waste clearance in brain. First, targeting of α-syntrophin to endfoot membranes is the most important factor determining the size of the perivascular AQP4 pool. Second, those regions that show high densities of α-syntrophin in endfoot membranes also show high levels of *Aqp4* transcripts. Third, regional heterogeneities in *Aqp4* transcript levels persist after α-syntrophin deletion. While important in their own right, these observations point to the existence of a regulatory factor, upstream of α-syntrophin expression, that controls the size of the perivascular AQP4 pool. The identification of this factor stands as an important goal for future studies.

## References

[CR1] Adams ME, Kramarcy N, Krall SP, Rossi SG, Rotundo RL, Sealock R, Froehner SC (2000). Absence of alpha-syntrophin leads to structurally aberrant neuromuscular synapses deficient in utrophin. J Cell Biol.

[CR2] Alvestad S, Hammer J, Hoddevik EH, Skare O, Sonnewald U, Amiry-Moghaddam M, Ottersen OP (2013). Mislocalization of AQP4 precedes chronic seizures in the kainate model of temporal lobe epilepsy. Epilepsy Res.

[CR3] Amiry-Moghaddam M, Ottersen OP (2003). The molecular basis of water transport in the brain. Nat Rev Neurosci.

[CR4] Amiry-Moghaddam M (2003). An alpha-syntrophin-dependent pool of AQP4 in astroglial end-feet confers bidirectional water flow between blood and brain. Proc Natl Acad Sci USA.

[CR5] Amiry-Moghaddam M (2003). Delayed K+ clearance associated with aquaporin-4 mislocalization: phenotypic defects in brains of alpha-syntrophin-null mice. Proc Natl Acad Sci USA.

[CR6] Amiry-Moghaddam M (2004). Alpha-syntrophin deletion removes the perivascular but not endothelial pool of aquaporin-4 at the blood–brain barrier and delays the development of brain edema in an experimental model of acute hyponatremia. FASEB J: Off Publ Fed Am Soc Exp Biol.

[CR7] Benfenati V, Caprini M, Dovizio M, Mylonakou MN, Ferroni S, Ottersen OP, Amiry-Moghaddam M (2011). An aquaporin-4/transient receptor potential vanilloid 4 (AQP4/TRPV4) complex is essential for cell-volume control in astrocytes. Proc Natl Acad Sci USA.

[CR8] Berod A, Hartman BK, Pujol JF (1981). Importance of fixation in immunohistochemistry: use of formaldehyde solutions at variable pH for the localization of tyrosine hydroxylase. J Histochem Cytochem.

[CR9] Binder DK, Oshio K, Ma T, Verkman AS, Manley GT (2004). Increased seizure threshold in mice lacking aquaporin-4 water channels. Neuroreport.

[CR10] Camassa LM (2015). Mechanisms underlying AQP4 accumulation in astrocyte endfeet. Glia.

[CR11] Culligan K, Glover L, Dowling P, Ohlendieck K (2001). Brain dystrophin-glycoprotein complex: persistent expression of beta-dystroglycan, impaired oligomerization of Dp71 and up-regulation of utrophins in animal models of muscular dystrophy. BMC Cell Biol.

[CR12] Dermietzel R (1973). Visualization by freeze-fracturing of regular structures in glial cell membranes. Naturwissenschaften.

[CR13] Eid T (2005). Loss of perivascular aquaporin 4 may underlie deficient water and K+ homeostasis in the human epileptogenic hippocampus. Proc Natl Acad Sci USA.

[CR14] Eilert-Olsen M (2012). Deletion of aquaporin-4 changes the perivascular glial protein scaffold without disrupting the brain endothelial barrier. Glia.

[CR15] Frigeri A, Nicchia GP, Nico B, Quondamatteo F, Herken R, Roncali L, Svelto M (2001). Aquaporin-4 deficiency in skeletal muscle and brain of dystrophic mdx mice. FASEB J: Off Publ Fed Am Soc Exp Biol.

[CR16] Furman CS, Gorelick-Feldman DA, Davidson KG, Yasumura T, Neely JD, Agre P, Rash JE (2003). Aquaporin-4 square array assembly: opposing actions of M1 and M23 isoforms. Proc Natl Acad Sci USA.

[CR17] Gundersen GA, Vindedal GF, Skare O, Nagelhus EA (2013). Evidence that pericytes regulate aquaporin-4 polarization in mouse cortical astrocytes. Brain Struct Funct.

[CR18] Haj-Yasein NN (2012). Aquaporin-4 regulates extracellular space volume dynamics during high-frequency synaptic stimulation: a gene deletion study in mouse hippocampus. Glia.

[CR19] Hiroaki Y (2006). Implications of the aquaporin-4 structure on array formation and cell adhesion. J Mol Biol.

[CR20] Igarashi H, Suzuki Y, Kwee IL, Nakada T (2014). Water influx into cerebrospinal fluid is significantly reduced in senile plaque bearing transgenic mice, supporting beta-amyloid clearance hypothesis of Alzheimer’s disease. Neurol Res.

[CR21] Iliff JJ (2012). A paravascular pathway facilitates CSF flow through the brain parenchyma and the clearance of interstitial solutes, including amyloid beta. Sci Transl Med.

[CR22] Jo AO, Ryskamp DA, Phuong TT, Verkman AS, Yarishkin O, MacAulay N, Krizaj D (2015). TRPV4 and AQP4 channels synergistically regulate cell volume and calcium homeostasis in retinal muller glia. J Neurosci.

[CR23] Jung JS, Preston GM, Smith BL, Guggino WB, Agre P (1994). Molecular structure of the water channel through aquaporin CHIP. The hourglass model. J Biol Chem.

[CR24] Landis DM, Reese TS (1974). Arrays of particles in freeze-fractured astrocytic membranes. J Cell Biol.

[CR25] Lunde LK, Camassa LM, Hoddevik EH, Khan FH, Ottersen OP, Boldt HB, Amiry-Moghaddam M (2015). Postnatal development of the molecular complex underlying astrocyte polarization. Brain Struct Funct.

[CR26] Manley GT (2000). Aquaporin-4 deletion in mice reduces brain edema after acute water intoxication and ischemic stroke. Nat Med.

[CR27] Manley GT, Binder DK, Papadopoulos MC, Verkman AS (2004). New insights into water transport and edema in the central nervous system from phenotype analysis of aquaporin-4 null mice. Neuroscience.

[CR28] Michele DE (2002). Post-translational disruption of dystroglycan-ligand interactions in congenital muscular dystrophies. Nature.

[CR29] Mola MG (2016). The speed of swelling kinetics modulates cell volume regulation and calcium signaling in astrocytes: a different point of view on the role of aquaporins. Glia.

[CR30] Nagelhus EA, Ottersen OP (2013). Physiological roles of aquaporin-4 in brain. Physiol Rev.

[CR31] Nakayama S, Migliati E, Amiry-Moghaddam M, Ottersen OP, Bhardwaj A (2016). Osmotherapy with hypertonic saline attenuates global cerebral edema following experimental cardiac arrest via perivascular pool of aquaporin-4. Crit Care Med.

[CR32] Neely JD, Amiry-Moghaddam M, Ottersen OP, Froehner SC, Agre P, Adams ME (2001). Syntrophin-dependent expression and localization of aquaporin-4 water channel protein. Proc Natl Acad Sci USA.

[CR33] Nicchia GP, Cogotzi L, Rossi A, Basco D, Brancaccio A, Svelto M, Frigeri A (2008). Expression of multiple AQP4 pools in the plasma membrane and their association with the dystrophin complex. J Neurochem.

[CR34] Nicchia GP, Rossi A, Nudel U, Svelto M, Frigeri A (2008). Dystrophin-dependent and -independent AQP4 pools are expressed in the mouse brain. Glia.

[CR35] Nielsen S, Nagelhus EA, Amiry-Moghaddam M, Bourque C, Agre P, Ottersen OP (1997). Specialized membrane domains for water transport in glial cells: high-resolution immunogold cytochemistry of aquaporin-4 in rat brain. J Neurosci: Off J Soc Neurosci.

[CR36] Papadopoulos MC, Verkman AS (2013). Aquaporin water channels in the nervous system. Nat Rev Neurosci.

[CR37] Papadopoulos MC, Manley GT, Krishna S, Verkman AS (2004). Aquaporin-4 facilitates reabsorption of excess fluid in vasogenic brain edema. FASEB J.

[CR38] Pisani F, Rossi A, Nicchia GP, Svelto M, Frigeri A (2011). Translational regulation mechanisms of aquaporin-4 supramolecular organization in astrocytes. Glia.

[CR39] Promeneur D, Lunde LK, Amiry-Moghaddam M, Agre P (2013). Protective role of brain water channel AQP4 in murine cerebral malaria. Proc Natl Acad Sci USA.

[CR40] Puwarawuttipanit W (2006). Differential effect of alpha-syntrophin knockout on aquaporin-4 and Kir4.1 expression in retinal macroglial cells in mice. Neuroscience.

[CR41] Rash JE, Staehelin LA, Ellisman MH (1974). Rectangular arrays of particles on freeze-cleaved plasma membranes are not gap junctions. Exp Cell Res.

[CR42] Rash JE, Yasumura T, Hudson CS, Agre P, Nielsen S (1998). Direct immunogold labeling of aquaporin-4 in square arrays of astrocyte and ependymocyte plasma membranes in rat brain and spinal cord. Proc Natl Acad Sci USA.

[CR43] Rossi A, Pisani F, Nicchia GP, Svelto M, Frigeri A (2010). Evidences for a leaky scanning mechanism for the synthesis of the shorter M23 protein isoform of aquaporin-4: implication in orthogonal array formation and neuromyelitis optica antibody interaction. J Biol Chem.

[CR44] Rurak J, Noel G, Lui L, Joshi B, Moukhles H (2007). Distribution of potassium ion and water permeable channels at perivascular glia in brain and retina of the Large(myd) mouse. J Neurochem.

[CR45] Sorbo JG, Moe SE, Holen T (2007). Early upregulation in nasal epithelium and strong expression in olfactory bulb glomeruli suggest a role for aquaporin-4 in olfaction. FEBS Lett.

[CR46] Thrane AS (2011). Critical role of aquaporin-4 (AQP4) in astrocytic Ca^2+^ signaling events elicited by cerebral edema. Proc Natl Acad Sci USA.

[CR47] Vajda Z (2002). Delayed onset of brain edema and mislocalization of aquaporin-4 in dystrophin-null transgenic mice. Proc Natl Acad Sci USA.

[CR48] Vajda Z, Pedersen M, Doczi T, Sulyok E, Nielsen S (2004). Studies of mdx mice. Neuroscience.

[CR49] Verbavatz JM, Ma T, Gobin R, Verkman AS (1997). Absence of orthogonal arrays in kidney, brain and muscle from transgenic knockout mice lacking water channel aquaporin-4. J Cell Sci.

[CR50] Yang B, Brown D, Verkman AS (1996). The mercurial insensitive water channel (AQP-4) forms orthogonal arrays in stably transfected Chinese hamster ovary cells. J Biol Chem.

[CR51] Yang J (2011). Loss of astrocyte polarization in the tg-ArcSwe mouse model of Alzheimer’s disease. J Alzheimer’s Dis: JAD.

[CR52] Yukutake Y, Yasui M (2010). Regulation of water permeability through aquaporin-4. Neuroscience.

[CR53] Yukutake Y (2008). Mercury chloride decreases the water permeability of aquaporin-4-reconstituted proteoliposomes. Biol Cell.

[CR54] Zelenina M (2010). Regulation of brain aquaporins. Neurochem Int.

[CR55] Zeynalov E, Chen CH, Froehner SC, Adams ME, Ottersen OP, Amiry-Moghaddam M, Bhardwaj A (2008). The perivascular pool of aquaporin-4 mediates the effect of osmotherapy in postischemic cerebral edema. Crit Care Med.

